# MicroRNA-98 interferes with thrombospondin 1 expression in peripheral B cells of patients with asthma

**DOI:** 10.1042/BSR20170149

**Published:** 2017-08-31

**Authors:** Liming Chen, Jianfeng Xu, Xiaoxia Chu, Chenghua Ju

**Affiliations:** Department of Hematology, Yantai Yuhuangding Hospital, Yantai 264000, China

**Keywords:** B lymphocyte, IL-10, microRNA

## Abstract

Thrombospondin 1 (TSP1)-producing B cells are an important immune regulatory cell fraction in the body, which are compromised in a number of immune diseases. miRs are involved in the immune regulation. The present study aims to elucidate the mechanism by which *miR-98* interferes with the expression of TSP1 in B cells of the peripheral blood system. In the present study, peripheral blood samples were collected from patients with allergic asthma. The B cells were isolated from the blood samples to be analyzed for the expression of *miR-98* and TSP1. The results showed that the levels of *miR-98* were higher, the levels of TSP1 were lower, in B cells isolated from the peripheral blood in patients with asthma. A negative correlation was identified between the data of *miR-98* and TSP1 in B cells. Exposure to T helper (Th) 2 (Th2) cytokine, interleukin (IL)-13, increased the expression of *miR-98* and suppressed the expression of TSP1 in peripheral B cells, which was abolished by knocking down the *miR-98* gene. In conclusion, *miR-98* can suppress the expression of TSP1 in the peripheral B cells of patients with allergic asthma.

## Introduction

A fraction of B cells in the peripheral blood system expresses thrombospondin 1 (TSP1). These B cells have the immune suppressive function on other effector immune cells’ activities [1]. TSP1^+^ B cells play an important role in the maintenance of the homeostasis in the body [2,3]. Published data indicate that the reduced number or the functional compromise of TSP1^+^ cell is associated with a number of immune disorders, such as allergic asthma [4], food allergy [5], and some other atopic diseases [6]. Factors or mechanisms leading to TSP1^+^ cell dysfunction are not fully understood yet.

Asthma is an allergic disease. Patients with asthma are usually sensitized to airborne antigens, such as house dust mites, pollens, animal skin debris etc [7]. The immune pathological feature of asthma is a T helper (Th) 2 (Th2) polarization condition in the airway mucosa, in which Th2 cells overproliferate and overproduce Th2 cytokines, including interleukin (IL)-4, IL-5, and IL-13 [8]. Th2 polarization facilitates the overproduction of antigen-specific immunoglobulin (Ig) E [9], which binds the high affinity receptor of IgE on the surface of mast cells to make the mast cells sensitized [10]. Upon re-exposure to specific antigens, the sensitized mast cells are activated to release preformed chemical mediators, such as histamine, to initiate allergic responses and the airway inflammation [11]. Although the studies on pathological feature of airway allergy advanced rapidly in the past decades, the therapeutic remedies for allergic diseases are still limited [12]. The treatment of airway allergy is currently unsatisfactory, in fact [13]. Therefore, it is still necessary to further investigate into the underlying mechanisms of allergy.

It is reported that a number of miRs are associated with the pathogenesis of immune disorders [7]. MiRs are non-coding ssRNA chains with 18–22 nts in length. *MiR-98* is one of the miRs interfering with the expression of the immune regulatory molecule IL-10 in B cells [14], and thus compromises the immune regulatory capacity in the body. For example, overexpression of *miR-98* is associated with the B-cell-related immune inflammation [15]. Since deregulation of TSP1 is also found in compromising the B cells’ immune regulatory functions [1,16], we hypothesized that *miR-98* is an important factor in the regulation of TSP1 expression in B cells in patients with asthma. Therefore, we observed the role of *miR-98* in the regulation of TSP1 in B cells of asthma patients. The results showed that the expression of *miR-98* was higher in the peripheral B cells of asthma patients, which mediated the effects of IL-13 on suppression of TSP1 in B cells.

## Materials and methods

### Patients

Patients with seasonal allergic asthma were recruited into the present study in non-allergy seasons. The diagnosis of asthma was performed by the physicians in our hospital. All the patients were in the remission period without apparent asthma symptoms. The demographic data of the patients are presented in Table 1. Exclusion criteria included: using immune suppressors; had apparent asthma symptoms; had severe organ diseases; and cancer. In addition, 20 healthy persons were also recruited into the present study to be healthy controls. The experimental procedures were approved by the Human Ethic Committee at our hospital. Written informed consent was obtained from each subject.

**Table 1:** Demographic data of asthma patients

Item	Asthma	Healthy
Age	28.5 ± 11.3	27.8 ± 9.6
Weight	55.6 ± 13.5	56.9 ± 9.7
Height	157.3 ± 22.1	159.3 ± 18.5
Gender (M/F)	10/10	10/10
Asthma	20 (100%)	
	10 (50%)	
FVC (%-pred.)	85.3 ± 3.2*	101.1 ± 0.2
FEV1 (%-pred.)	95.6 ± 9.7*	99.8 ± 0.6
Total IgE (IU/l)	589.3 ± 187.5*	<0.35
Specific IgE (IU/l)	83.2 ± 13.9*	<0.35

The values are presented as mean ± S.D. *, *P*<0.01, compared with healthy subjects. Abbreviations: FEV1, forced expiratory volume in 1 s; FVC, forced vital capacity.

### Assessment of TSP1^+^ B cells in peripheral blood by flow cytometry

Approximately 30 ml peripheral blood was collected from each human subject via ulnar vein puncture. Peripheral blood mononuclear cells (PBMCs) were isolated from the blood samples by gradient density centrifugation. The PBMCs were stained with FITC-labeled anti-CD19 mAb or isotope IgG (BD Bioscience) for 30 min at 4°C, washed with PBS, fixed with 1% paraformaldehyde for 1 h, incubated with 0.5% saponin for 30 min, stained with APC-labeled anti-TSP1 mAb or isotope IgG (BD Bioscience) for 30 min at 4°C, again washed with PBS, and analyzed by flow cytometry (FACSCanto II, BDBioscience). The data were analyzed with software FlowJo (Tree Star). Data from isotope IgG staining were used as a gating reference.

### Assessment of *miR-98* and TSP1 by real-time quantitative RT-PCR

B cells were purified from PBMCs by magnetic cell sorting with a reagent kit (Miltenyi Biotech) following the manufacturer’s instructions. The purity of the isolated B cells was greater than 98% as checked by flow cytometry. The B cells were cultured in RPMI1640 medium supplemented with 10% FBS, 100 U/ml penicillin, 0.1 mg/ml streptomycin, 2 mM L-glutamine, and 20 ng/ml anti-CD40 mAb. Total RNA was extracted from the B cells with TRIzol reagent (Invitrogen). The cDNA was synthesized with the RNA and a reverse transcription kit (Invitrogen). The samples were amplified in a real-time PCR device (Bio–Rad) with SYBR Green Master Mix and the *miR-98* primers (gtgaggtagtaagttgtatt and ggaaagtagtaagttgtata) and the TSP1 primers (gcaagtcacccagtcctact and aatgaaacccgtctttggcc). The results were calculated by the 2^−ΔΔ*C*^_t_ method and presented as fold change against reference controls. The primers of housekeeping gene, β-actin are cgcaaagacctgtatgccaa and cacacagagtacttgcgctc. The primers were provided by the Enke Biotech (Shenzhen, China).

### Assessment of serum cytokine levels by ELISA

The sera were isolated from blood samples by centrifugation. The levels of IL-4, IL-5, IL-13, and interferon (IFN)-γ in the sera were determined by ELISA with purchased reagent kits (R&D Systems) following the manufacturer’s instructions.

### Test the role of *miR-98* in the suppression of TSP1 in B cells

To increase the expression of TSP1, B cells (10^6^ cells/ml) were stimulated with lipopolysaccharide (LPS; Sigma–Aldrich) at 1 µg/ml in the culture for 48 h. To test the effects of IL-13 on suppression of TSP1 in B cells, B cells were cultured in the presence of both LPS and IL-13 (200 ng/ml; R&D Systems) in the culture for 48 h. To test the role of *miR-98* in the IL-13-suppressed TSP1 expression in B cells, *miR-98*-deficient B cells and wild B cells were exposed to LPS and IL-13 in the culture for 48 h. To avoid B-cell apoptosis, anti-CD40 mAb (20 ng/ml; Santa Cruz Biotechnology) was added to the culture.

### Preparation of *miR-98*-deficient B cells by RNAi

B cells were transduced with *miR-98* shRNA-laden lentivirus or control lentivirus (Enke Biotech, Shenzhen, China) following the manufacturer’s instructions. Briefly, 1 µg RNAi reagents were added to 1×10^5^ B cells together with the transduction reagents provided by the reagent kits. Forty-eight hours after transduction, the effect of RNAi was assessed by real-time quantitative RT-PCR (RT-qPCR).

### Statistics

Data were normally distributed as analyzed by Norm.DIST software (Microsoft Excel). Data were processed with the GraphPad Prism 5 software. The difference between the two groups was determined with the Student’s *t* test or ANOVA if more than two groups. The Pearson correlation assay was used to determine the correlation between groups. *P*<0.05 was set as a significant criterion.

## Results

### The frequency of peripheral TSP1^+^ B cells is negatively correlated with *miR-98* expression

**Figure 1:**
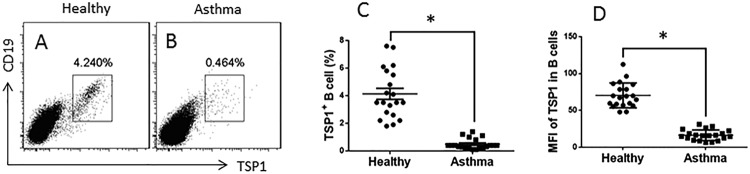
Evaluation of peripheral TSP1 expression B cells (**A**,**B**) The gated dot plots show representatives of peripheral TSP1^+^ B cells in PBMCs of healthy subjects (*n*=20) and asthma patients (*n*=20). (**C**) The dot plots show the individual data of TSP1^+^ B cell frequency of 20 healthy subjects and 20 asthma patients. (**D**) The mean fluorescence intensity of TSP1 staining in B cells (*t* test). *P<0.01, compared with the healthy group.

**Figure 2:**
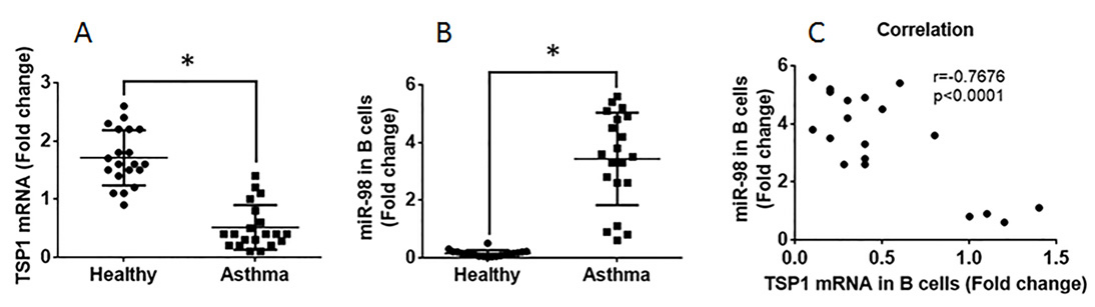
Assessment of *miR-98* in B cells (**A**) The individual value of *TSP1* mRNA in peripheral B cells of asthma patients (*n*=20) and healthy subjects (*n*=20). (**B**) The individual value of *miR-98* in peripheral B cells of asthma patients and healthy subjects. (**C**) The correlation between the frequency of TSP1^+^ B cells and the *miR-98* in the B cells (*t* test).

We first assessed the frequency of peripheral TSP1^+^ B cells in asthma patients. The results showed that the frequency of TSP1^+^ B cells (0.46%) was significantly less than that in healthy controls (4.24%; Figure 1). Since *miR-98* inhibits TSP1 expression [14], we isolated peripheral B cells to be analyzed by RT-qPCR. The results showed that the *TSP1* mRNA levels in B cells of patients with asthma were significantly lower than that in the healthy control group (Figure 2A), while the levels of *miR-98* in peripheral B cells were higher in the asthma group than the healthy group (Figure 2B). To assess if there is an association between TSP1^+^ B-cell frequency and the expression of *miR-98*, we performed a correlation assay with the data on peripheral TSP1^+^ B-cell frequency and the *miR-98* levels in B cells. The results showed a negative correlation (r = –0.7676, *P*=0.0001) between the frequency of peripheral B cells and the *miR-98* levels in B cells (Figure 2C).

### IL-13 suppresses TSP1 expression in B cells via up-regulating expression of *miR-98*

**Figure 3:**
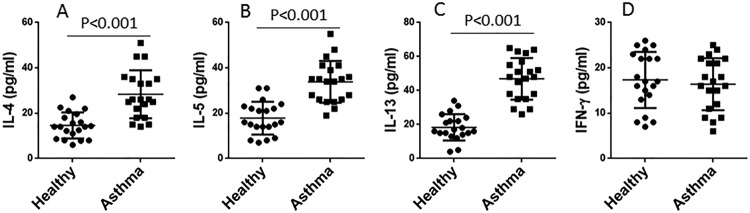
Assessment of serum cytokines These figures show the individual data of serum levels of IL-4 (**A**), IL-5 (**B**), IL-13 (**C**), and IFN-γ (**D**) from healthy subjects (*n*=20) and asthma patients (*n*=20) (*t* test).

Considering that serum cytokines might affect the expression of *miR-98* in B cells, we assessed the cytokine levels in the sera. The results showed that the Th2 cytokine levels were significantly higher in asthma patients than that in healthy controls (Figure 3A–C), while the Th1 cytokine, IFN-γ levels were not different between the two groups (Figure 3D).

**Figure 4:**
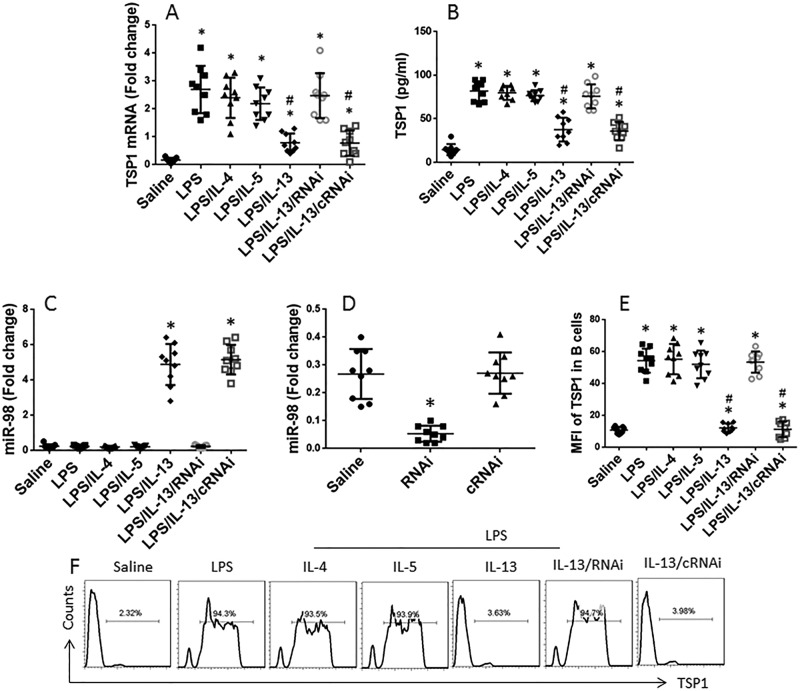
Assessment of TSP1 expression in B cells (**A**) The *TSP1* mRNA levels in B cells after the treatments as denoted on the *x*-axis in the culture for 48 h. (**B**) The TSP1 levels in the culture supernatant (by ELISA). (**C**) The *miR-98* levels in B cells after the treatment with the procedures denoted on the *x*- axis. (**D**) The *miR-98* levels in B cells after RNAi. RNAi: B cells were transduced with *miR-98* shRNA-laden lentivirus. cRNAi: B cells were transduced with control lentivirus. (**E**) The MFI (medium fluorescence intensity) of TSP1 in B cells. (**F**) The frequency of TSP1^+^ B cells after treatment denoted above each panel. The experiments were repeated three times with the same samples. Each sample was analyzed in triplicate. *, *P*<0.0001, compared with the saline group. ^#^, *P*<0.0001, compared with the LPS group (*t* test).

Data reported above implicate that Th2 cytokines might modulate the expression of TSP1 in B cells. To test this, we isolated B cells from healthy persons. The B cells were cultured in the presence of LPS together with IL-4, IL-5, or IL-13, in the culture for 48 h. As analyzed by RT-PCR and ELISA, exposure to LPS markedly up-regulated the expression of TSP1 in B cells, which was inhibited by exposure to IL-13, but not IL-4 or IL-5 (Figure 4A,B; *P*<0.01). Considering that *miR-98* might be involved in the IL-13 suppressed IL-10 expression in B cells, we measured the levels of *miR-98* in B cells. The results showed that exposure to IL-13 markedly increased the *miR-98* levels in B cells (Figure 4C). Next, we prepared *miR-98*-deficient B cells by transducing B cells with *miR-98* shRNA-laden lentivirus. The transduction resulted in approximately ten-folds down of the expression of *miR-98* in B cells (Figure 4D). Then, we treated the *miR-98*-deficient B cells with LPS and IL-13. The results showed that exposure to IL-13 did not block the TSP1 expression in the *miR-98*-deficient B cells (Figure 4A,B). The B cells were also analyzed by flow cytometry. The results showed that the mean fluorescence intensity and the frequency of TSP1^+^ B cells were in-line with the results of *TSP1* mRNA expression and TSP1 in the culture supernatant (Figure 4E,F).

## Discussion

TSP1 is a critical immune regulatory molecule [17]. TSP1^+^ B cells express TSP1 that can inhibit abnormal immune responses [1]. Thus, TSP1^+^ B cells are an important cell population in the maintenance of the homeostasis in the body. The present data show that much less TSP1^+^ B cells in the peripheral blood system of patients with asthma, suggesting that the reduction in peripheral TSP1^+^ B cells is one of the pathological features of allergic asthma. Other investigators also found a similar phenomenon. Liao et al. [18] found that TSP1^+^ B cells were less in asthma patients. Braza et al. [19] observed a novel fraction of CD9^+^ B cells expressing TSP1; adoptive transfer of CD9^+^ B cells inhibited airway inflammation and reversed lung function by inhibiting Th2- and Th17-pattern inflammation in TSP1-dependent manner, which restored immunological balance in lung tissues.

To find the causative factors for the reduction in TSP1^+^ B cells is of significance. Our data showed abnormally higher expression of *miR-98* in peripheral B cells. The levels of *miR-98* in peripheral B cells were negatively correlated with the frequency of peripheral TSP1^+^ B cells. The fact indirectly suggests that *miR-98* may be an important factor suppressing TSP1^+^ B cells in asthma patients. The inference was supported by the subsequent data. We found that Th2 cytokine IL-13 could suppress the expression of TSP1 in B cells, which was mediated by *miR-98* because knockdown of *miR-98* abolished the IL-13-suppressed TSP1 expression in B cells. Others also found that IL-13 suppressed the gene transcription in a large number of molecules via altering the epinenetic pathway [20]. Yang et al. [1] reported that exposure to IL-13 inhibited TSP1 expression in B cells via increasing the TSP1 gene methylation. Whether IL-13 represses TSP1 expression also via the epigenetic pathway is to be further investigated.

Asthma is a disease induced by multiple factors. Allergy is the major causative factor in the pathogenesis of asthma. The present study focusses on the role of *miR-98* in the regulation of IL-10 expression in B cells of patients with allergic asthma. It is well documented that allergic asthma patients show high levels of Th2 cytokines in the serum, infiltration of eosinophils in the lung as shown in the sputum, low frequency of regulatory B cells and less regulatory T cells [21]. These immune pathological features demonstrate that the immune regulatory functions in allergic asthma patients are compromised [22]. The present study found that the expression of *miR-98* was increased and TSP1 was decreased in peripheral B cells of allergic asthma patients, indicating that the *miR-98* may be a novel causative factor contributing to the pathogenesis of asthma.

The data show that the expression of TSP1 in peripheral B cells is lower in asthma patients than in healthy subjects. TSP1 has an immune regulatory function and contributes to the maintenance of immune homeostasis in the body [23]. Deficiency of TSP1 may suffer from immune disorders [24]. To generate TSP1 expression in B cells also contributes to the therapeutic effects of allergen-specific immunotherapy [16]. The present study revealed one of the causative factors of TSP1 expression in B cells that *miR-98* could suppress the expression in B cells, indicating that to modulate the expression of *miR-98* may improve the treatment of asthma, which needs to be further investigated.

In summary, the present data show that much less frequency of TSP1^+^ B cells was found in the peripheral blood system in patients with asthma. *MiR-98* played a critical role in the IL-13-suppressed TSP1 expression in B cells. Blocking *miR-98* abolished the IL-13-suppressed TSP1 expression.

## Abbreviations

IFN: interferon
IL: interleukin
LPS: lipopolysaccharide
PBMC: peripheral blood mononuclear cell
RT-qPCR: real-time quantitative RT-PCR
Th2: T helper 2
TSP1: thrombospondin 1
